# Synthesis and Ex Vivo Trans-Corneal Permeation of Penetratin Analogues as Ophthalmic Carriers: Preliminary Results

**DOI:** 10.3390/pharmaceutics12080728

**Published:** 2020-08-03

**Authors:** Silvia Pescina, Marina Sala, Maria Carmina Scala, Patrizia Santi, Cristina Padula, Pietro Campiglia, Carmine Ostacolo, Sara Nicoli

**Affiliations:** 1 Department of Food and Drug, University of Parma, Parco Area delle Scienze 27/a, 43124 Parma, Italy; patrizia.santi@unipr.it (P.S.); cristina.padula@unipr.it (C.P.); sara.nicoli@unipr.it (S.N.); 2 Department of Pharmacy, University of Salerno, via G. Paolo II 132, Fisciano, 84084 Salerno, Italy; msala@unisa.it (M.S.); mscala@unisa.it (M.C.S.); pcampiglia@unisa.it (P.C.); 3 Department of Pharmacy, University of Napoli Federico II, via D. Montesano 49, 80131 Napoli, Italy; ostacolo@unina.it

**Keywords:** penetratin, CPP, trans-corneal, ex vivo, enhancer, ocular delivery

## Abstract

Among enhancing strategies proposed in ocular drug delivery, a rising interest is directed to cell penetrating peptides (CPPs), amino acid short sequences primarily known for their intrinsic ability to cell internalization and, by extension, to cross biological barriers. In fact, CPPs may be considered as carrier for delivering therapeutic agents across biological membranes, including ocular tissues. Several CPPs have been proposed in ophthalmic delivery, and, among them, penetratin (PNT), a 16-amino acids natural peptide, stands out. Therefore, we describe the synthesis via the mimotopic approach of short fluorescently labeled analogues of both PNT and its reversed sequence PNT-R. Their ability to cross ocular membranes was checked ex vivo using freshly explanted porcine cornea. Furthermore, some sequences were studied by circular dichroism. Despite the hydrophilic nature and the relatively high molecular weight (approx. 1.6 kDa), all analogues showed a not negligible trans-corneal diffusion, indicating a partial preservation of penetration activity, even if no sequences reached the noteworthy ability of PNT. It was not possible to find a correlation between structure and corneal penetration ability, and further studies, exploring peptides distribution within corneal layers, for example using imaging techniques, deserve to be performed to figure out a possible difference in intracellular delivery.

## 1. Introduction

Ophthalmic administration, despite the large availability of many effective drugs, is still challenging for both the anterior and the posterior segments of the eye, due to several types of barriers, exerting static, dynamic and metabolic resistance [1]. Topical non-invasive approach, mainly addressing the anterior segment, and intraocular injections, the first choice for a productive delivery to the back of the eye, are characterized by different drawbacks. Particularly, the low bioavailability of topically applied drugs, responsible for high frequency of administration, as well as the high invasiveness of the injective routes, explain the significant number of unsuccessful treatments [2].

Several delivery strategies have been developed and many of them are currently available on the market: among others, solid devices (ocular inserts for topical use and ophthalmic implants to be intravitreally administered) allowing for a sustained release of the drug over time; nanosystems (like micelles, liposomes, nanoparticles); physical enhancers, like iontophoresis and ultrasounds [3]. Recently, there has been an increasing emphasis on cell penetrating peptides (CPPs), short amino acid sequences (usually less than 30 amino acids), natural (i.e., tat from human immunodeficiency virus-1; penetratin (PNT), from antennapedia of *Drosophila melanogaster*) or synthetic (i.e., MAP; GALA), commonly cationic (i.e., TAT, PNT) [4]. A key feature of CPPs, as suggested by their name, is the intrinsic ability to enter cells by translocating across cellular membrane and therefore, they have been proposed as carriers for therapeutic compounds. This implies not only cell internalization, but also the possibility to develop CPPs carrying therapeutics, covalently or not-covalently bound, across biological membranes, including ocular membranes. As mentioned, since ocular membranes hinder the drug diffusion, CPPs have been suggested as possible carrier for both anterior and posterior targeting [5]. Particularly, anterior segment of the eye might benefit from CPPs application for a more effective treatment of diseases affecting cornea, but also the anterior chamber and the lens. To explore this perspective, following the mimotopic approach, we previously designed and prepared short PEP-1 derived CPPs. The mimotopic approach allowed us to obtain shortened PEP-1 sequences, more affordable and with a comparable penetration capacity. Nevertheless, once applied ex vivo to porcine cornea, all PEP-1 analogues and PEP-1 itself, were not able to match PNT, chosen as reference CPP, demonstrating its superiority in both permeation across and distribution within corneal layers. Moreover the synthesized peptides and PEP-1 itself showed a preferential paracellular penetration route in comparison with the intracellular penetration route of PNT [6]. Therefore, according to these evidences, the aim of the present paper is the design and synthesis of new PNT analogues by the mimotopic approach and the study of their capability to cross porcine cornea ex vivo.

In particular, we systematically examine the structural consequences of transformation of the parent sequence (PNT) considering its reversed sequence (PNT-R). Retro-isomers are generally considered peptides with similar physico-chemical (length, net charge and proportion of hydrophilic and hydrophobic amino acids) and structural properties compared to their partners; the direction of the termini and peptide bonds are reversed. It has been reported that reversing the sequence impacts on the lateral surface stability of peptide monolayer and gives a differential way of peptide/lipid interaction at the presence of model membranes [7]. All these features have been correlated with the different efficacy of CPPs [8,9]. For these reasons, using a mimotopic approach, we synthesized an overlapping library of peptides and their retro-isomers to generate a panel of sequences of specific length and offset to cover the entire native sequence.

All new synthesized PNT analogues were labelled with 5,6 carboxyfluorescein (FAM), to make them fluorescent. The glycyl–glycine spacer (GG) was introduced between PNT analogues sequence and FAM in order to minimize steric hindrance between the bulky fluorescent probe and the primary structure of CPP and, consequently, to preserve their secondary structure. Finally, the effect of the physico-chemical properties of the fluorescent probe was investigated by replacing caboxyfluorescein with the more hydrophobic fluorescein. Synthesized peptides were challenged for their ability to permeate across the cornea using an ex vivo validated assay.

## 2. Materials and Methods

### 2.1. Materials

Coupling agents (HOAt, HOBt, HBTU), Nα-Fmoc-protected amino acids, DIEA, piperidine, and trifluoroacetic acid were provided by Iris Biotech (Marktredwitz, Germany). Peptide synthesis was performed using a Rink Amide-ChemMatrix resin (Biotage AB, Uppsala, Sweden). All other solvents and reagents were of analytical grade, commercially available and were used without further purification unless otherwise noted. 5(6)-Carboxyfluorescein (FAM; mixture of isomers, 97%; MW 376.3 g/mol) and fluorescein (FL; MW 332.31 g/mol) were from Merck (Darmstadt, Germany), while bovine serum albumin (BSA) and dimethylsulfoxide (DMSO) were purchased from Sigma Aldrich (St. Louis, MO, USA). Phosphate buffered saline (PBS) was prepared by dissolving 0.19 g/L KH_2_PO_4_, 2.37 g/L Na_2_HPO_4_, 8.8 g/L NaCl in pure water (Purelab^®^ Pulse, ELGA LabWater, High Wycombe, UK); final pH was adjusted to 7.4 with H_3_PO_4_ (85 wt. %).

### 2.2. Microwave Peptide Synthesis

The synthesis of all peptides (Table 1) was performed with a solid phase approach using a standard Fmoc methodology on a Biotage Initiator + Alstra automated microwave synthesizer (Biotage, Uppsala, Sweden).

Peptides were synthesized on Rink-Amide-ChemMatrix resin (0.150 g, loading 0.3 mmol/g). Coupling reactions were performed using Nα-Fmoc amino acids (4.0 eq., 0.5 M), HBTU (3 eq, 0.6 M), HOAt (3 eq, 0.5 M), and DIEA (6 eq, 2 M) in N-methyl-2-pyrrolidone (NMP) for 10 min at 75 °C (2×). After each coupling step, the Fmoc protecting group was removed with 25% piperidine/DMF (1× 3 min, 1 × 10 min) at room temperature. The resin was washed with DMF (4 × 4.5 mL) after each coupling and deprotection step. Finally, the N-terminal Fmoc group was removed, resin-bound peptides were coupled with 5(6)-carboxyfluorescein and fluorescein using Dic (6eq) Hobt (3 eq) for 20 min at 60 °C. Completeness of *N*-terminal acylation was confirmed using the Kaiser test. The resin was washed with DCM (7×), and the peptide released from the resin with TFA/TIS/H_2_O (ratio 90:5:5) for 3 h. The resin was removed by filtration and the crude peptide recovered by precipitation with cold anhydrous ethyl ether to give a yellowish powder that was then lyophilized.

Finally, peptides were purified and characterized by RP-HPLC and mass spectrometry as previously reported [6]. Analytical data are shown in Supplementary Materials (Table S1 and Figures S1–S15).

### 2.3. Trans-Corneal Permeation Studies

Permeation experiments across freshly porcine cornea were performed following a method previously reported [11]. In detail, transparent porcine corneas were isolated from fresh pig eyes (breed: Large White and Landrace; weight: 145–190 kg; age: 10–11 months; sex: female and male; Macello Annoni SpA, Busseto, Italy) and supported by glass Franz-type diffusion cells, having a permeation area of 0.2 cm^2^. The epithelial side was in contact with the donor, i.e., 200 µL of aqueous solution containing a peptide: each concentration and solvent are indicated in Table 2. Donor concentrations were selected based on different solubility and analytical sensitivity (see paragraph 2.4 and Table S2). For some peptides, the solubilization was improved by dissolving the raw material using small amount of DMSO (final concentration less than 5% v/v), and then PBS or PBS+0.1% BSA were alternately added.

To fill the receptor compartment, around 4 mL of degassed PBS or PBS+0.1% BSA was used (see Table 2), being kept at 37 °C and maintained under magnetic stirring to avoid any boundary layer effect. The presence of 0.1% BSA aids in preventing peptides aggregation. Permeation experiments lasted 6 h, and for quantification purposes, 200 µL of the receiving solution were collected every hour and reintegrated with fresh medium. Each condition was replicated between 3 and 4 times, using cornea isolated from different animals. In the present work, blank experiments were not performed, since the absence of tissue interference has already been demonstrated [6].

### 2.4. Analytical Method

Permeation samples were analysed without preliminary separation by fluorescence, using a Spark 10M microplate reader (Tecan Trading AG, Switzerland); excitation and emission wavelengths were 485 nm and 535 nm, respectively. Standard solutions were prepared in PBS pH 7.4 or in PBS containing BSA 0.1%. RSD% (relative standard deviation %) was lower than 10%, while ER% (relative error %) resulted lower than 15%. RSD% and ER% values for each peptide, as well as intervals of calibration curves, are reported in Table S2.

### 2.5. Circular Dichroism (CD) Measurements

All CD spectra were recorded using a JASCO J810 spectropolarimeter at 258C in the range λ 260–190 nm (1 mm path length, 1 nm bandwidth, four accumulations, and a scanning speed of 10 nm/min). Measurements were performed with peptides in PBS (0.100 mM, pH 7.4) or in 40% hexafluoroisopropanol (HFIP)/PBS solution. Spectra were corrected for the solvent contribution. Estimation of secondary structure content was performed using the algorithms CONTIN and SELCON from the DichroWeb website: http://dichroweb. cryst.bbk.ac.uk/html/home.shtml [12].

### 2.6. Data Processing

The amount of CPP permeated across the cornea (nmol/cm^2^) is plotted against time (hour): the slope of the regression line at steady state represents the trans-corneal flux J (nmol/cm^2^ h). The apparent permeability coefficient P*_app_* (cm/s) is then calculated as Equation (1):

P*_app_* = J/c (1)

being c (µM) the concentration of the donor solution. P*_app_* allows for a direct comparison among permeants studied using different donor concentrations.

### 2.7. Statistical Analysis

Data were reported as mean ± sd, unless otherwise noted. The differences between values was assessed using Student’s t test and considered statistically significant when *p* < 0.05.

## 3. Results and Discussion

Penetratin is a 16-amino acids amphipathic CPP of natural origin, very effective in cell internalization, by both direct translocation and endocytosis. For this reason, it is extensively investigated in different fields of drug delivery, such as brain, skin, intestinal wall [13] and, more recently, ocular. In fact, PNT has been proposed for ophthalmic application by Liu and collaborators, who successfully demonstrated its ability to reach the posterior segment of the eye after topical administration in vivo using rats [14], while some Authors described the use of penetratin to functionalize dendrimer-based nanoparticles intended for a non-invasive treatment of the back of the eye [15,16]. The ability to trans-corneally diffuse of PNT, and more in general of CPPs, appears relevant not only in view of a non-invasive delivery to the back of the eye, but even to address the diseases affecting the anterior segment (i.e., cornea, anterior and posterior chambers and lens), as suggested by different Authors [17,18]. In this regard, we reported a study of ex vivo diffusion and distribution inside cornea of peptides deriving from PEP-1, a synthetic CPP [6]; in the present work our attention was focused on PNT analogues. Particularly, shorter cationic peptides have been synthesized, reducing the number of residues of the primary structure (Table 1), thus improving chemical accessibility and highlighting some structure-activity relationship clues. Moreover, it has been reported how shorter oligomers as CPPs are characterized by improved solubility, protease resistance, and lower toxicity in comparison with their longer congeners [19].

**Figure 1:**
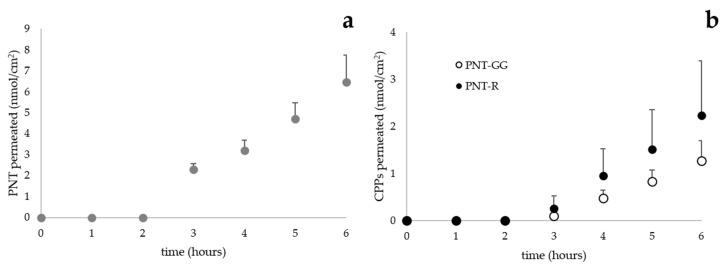
Trans-corneal permeation profiles of PNT (donor solution 100 µM; panel (**a**)) and PNT-GG and PNT-R (donor solution 25 µM; panel (**b**)). (mean value ± sem).

PNT, considered as reference sequence, showed a permeation profile (Figure 1a) and an apparent permeability coefficient (Table 2) comparable to those obtained in our previous work.

**Figure 2:**
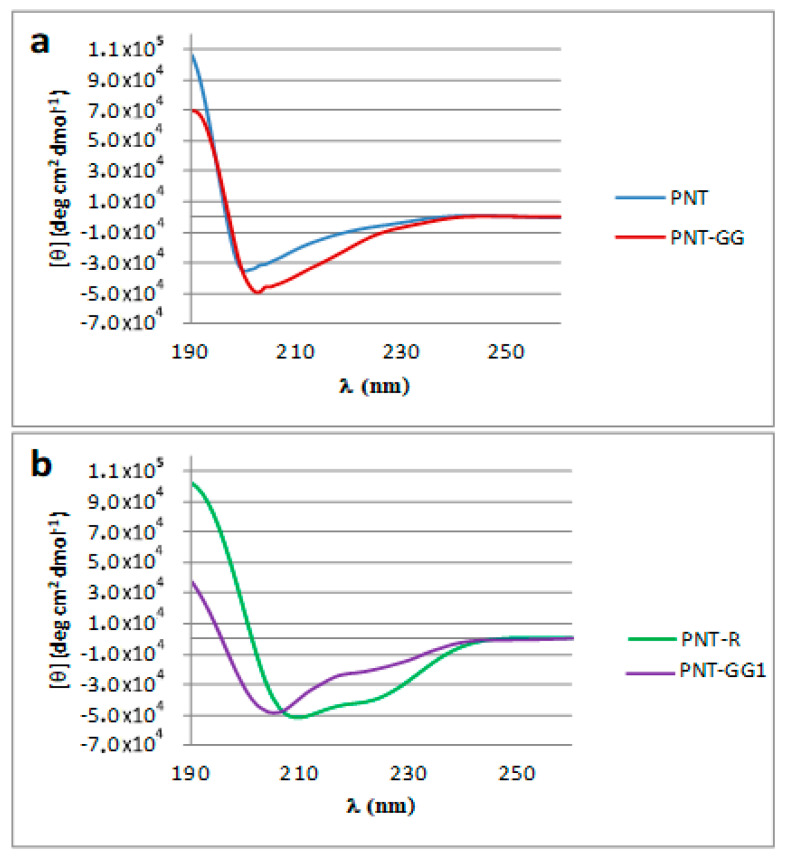
CD spectra for PNT (blue line), PNT-GG (red line) (**a**) and PNT-R (green line), PNT-GG 1 (purple line) (**b**) in 40% HFIP/PBS solution.

In PNT-GG and PNT-R a glycine-glycine spacer has been added to avoid steric hindrance between the fluorescent probe and the primary structure of CPP preserving their secondary structure [6]: as shown in Figure 1b, the trans-corneal permeation profiles are overimposable. Despite the presence of the spacer, the CD spectra of PNT and PNT-GG (Figure 2) did not reveal a difference in the secondary structure of the peptides in the hydrophobic environment.

The circular dichroism spectra were, in fact, acquired in PBS buffer or in hexafluoroisopropanol (HFIP)/PBS solution, at a peptide concentration of 100 μM and recorded at 25 °C, to gauge the effect of environment on secondary structure formation. In aqueous solution, a random coil structure is favored for both peptides (data not shown), while in aqueous solution containing 40% HFIP (Figure 2) they both adopt a comparable percentage of alpha helix and beta strand.

On the other hand, the retro-isomer PNT-R showed a difference in the secondary structure in hydrophobic environment. The presence of HFIP, in fact, promoted the transition from a random coil to an alpha—helical structure as witnessed by the weak minima at 208 and 220 nm (Figure 2). So, an alpha helix content is shown for all peptides but to a much lesser extent for the PNT and PNT-GG; CD analysis in fact reveals a 98% alpha-helicity for PNT-R vs. 10% helicity of PNT and PNT-GG. Unexpectedly, these differences did not influence the trans-corneal diffusion, since there are no statistical differences between PNT, PNT-GG and PNT-R in permeability (Table 2). Furthermore, we have deepened our knowledge on the secondary structure of peptides that do not show permeability, to observe if any structural differences could influence permeability. The study was carried out on PNT-GG 1 (as non-permeable model peptide). As shown in Figure 2b, PNT-GG 1 showed increased helicity than PNT-GG and PNT (20% of alpha helix structure) but, despite the presence of the cell membrane translocation sequence, the peptide is unable to properly work as CPP (Table 2).

**Figure 3:**
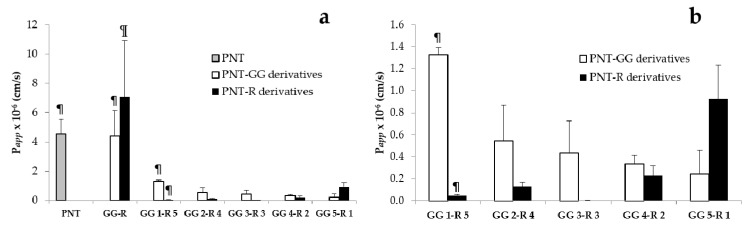
(**a**) Permeability coefficients of PNT and analogues. (**b**) Magnification of permeability coefficients of decapeptides. (¶ indicates the presence of translocation fragment KKWKMRR; PNT in grey, PNT-GG derivatives in white, PNT-R derivatives in black; mean value ± sem).

Ex vivo permeation experiments were then performed starting from solutions of PNT and its analogues in the concentration range between 25 and 250 µM: these values have been selected considering solubility and analytic sensitivity. Trans-corneal permeation of all shorter decapeptides was always lower than PNT and PNT-R or PNT-GG, respectively (Table 2; Figure 3a) and their apparent permeability were not in correlation either with mass (molecular weight approx. 1600 g/mol), or with charge (from +2 to +5).

However, data are affected by a high variability (Table 2) that can be ascribed to the propensity of the peptides to self-aggregate in aqueous solution. These aggregates, although macroscopically unobservable, may significantly impact on both the penetration properties and the CPP detection, because of the high sensitivity of the fluorescence technique [20]. In order to limit the self-aggregation, 0.1% BSA was added to PBS, but the result did not significantly improve. In addition, the variability of the apparent permeability coefficients, may also be due to the intrinsic variability of the tissues, that can have a more pronounced impact in case of high molecular weight compounds [21].

Despite the lower performance of the newly synthetized peptides with respect to PNT, the obtained results could contribute to raise awareness and understanding in the field of CPP-mediated ocular delivery. In fact, although a constant increasing in data availability is occuring, demonstrated by the high number of papers published in the last years, the knowledge of the penetration mechanisms is still incomplete [5,22]. Each CPP is a unique entity and both the cargo (drug or fluorophore) and the surrounding biological environment (type of cell, experimental conditions, etc.) might significantly affect its final behavior. Therefore, the comparison between new results and literature data appears quite challenging. Particularly, considering our outcomes, some remarks are possible. Fischer and collaborators prepared analogues, covalently linked with biotinyl group, and studied their internalization in cells [10]. Amongst other sequences, two correspond to PNT-GG 1 and PNT-GG 5, respectively. Although the experiment was conducted on cells and peptides carried a different cargo, the behavior of the two Fischer sequences appears comparable to our analogues. In fact, the internalization of the sequence corresponding to PNT-GG 5 is reported to be between 10 and 20% with respect to penetratin, while the peptide corresponding to PNT-GG 1 shows a translocation ability of 60%. By comparing the apparent permeability coefficients obtained in the present work, a qualitatively similar result is observed, since PNT-GG 1 shows a better diffusive performance than PNT-GG 5 (*p* < 0.01) and both are less performing than PNT. This evidence suggests the relevance of the transcription fragment KKWKMRR, that is the C terminal fragment responsible for cell translocation, as demonstrated by using human fibroblast and lung cancer cell lines [10]. In fact, the sequence KKWKMRR is absent in PNT-GG 5, but contained in PNT-GG 1, as well as in PNT, PNT-GG and PNT-R (Table 1). However, the presence of the transcription fragment is not always predictable of successful peptides: this sequence is also contained in PNT-R 5, the retro-isomers of PNT-GG 1, that in contrast showed a very low trans-corneal permeability (P*_app_* PNT-GG 1 vs. PNT-R 5 *p* < 0.01; Figure 3b, Table 2). Similarly, the behavior of PNT-GG 1 was not statistically different from PNT-R 1, the reciprocal peptide of PNT-GG 5, which is lacking transcription fragment.

We have also analysed if the sequence reciprocity (GG vs. R) plays a role in the diffusivity of the peptide across the cornea and we found no difference between most of the paired sequences, namely PNT-GG 2 vs. PNT-R 4, PNT-GG 3 vs. PNT-R 3, PNT-GG 4 vs. PNT-R 2, PNT-GG 5 vs. PNT-R 1 (Figure 3b). This confirms that the sequence is only one of the factors affecting the translocation: in fact, even CPP concentration, type of cells, and cargo can strongly influence CPP final behavior/activity [23]. Particularly, data highlight a strong influence of the cargo on the peptide characteristics: when the cargo was represented by fluorescein (FL), obtained peptides (PNT-R-FL and PNT-R-FL 4; Table 1) were poorly soluble in aqueous medium and for this reason they have not been used in ex vivo permeation experiments. Differently, the presence of FAM, a more hydrophilic fluorescent probe, allowed for aqueous solutions ranging in concentration from 25 to 250 µM (Table 2). No experiments were conducted above this concentration since both PNT-GG and PNT-R, despite undergoing complete solubilization at 250 µM, after two hours in contact with cornea gave the formation of yellowish precipitates that were not given by PNT solutions at the same concentration.

The lack of correspondence between peptides secondary structure and their membrane permeation ability has been previously reported for some CPPs characterized by inversion of chirality at specific positions, despite that D-amino acids are known to destabilize α-helices [24,25]. Nevertheless, it is well known that ion pair interaction between the arginine residues of penetratin and anionic head groups, like heparin sulphate, plays a pivotal role during the early stages of membrane translocation [26]. This interaction is accounted for in the CPPs transition from random coil to an organized secondary structure [27]. Moreover, it should be considered that, efficient CPPs, after membrane interaction, should move from the water environment, a high dielectric medium, to the membrane core, a really low dielectric medium. Thus, CPPs hydrophobic regions assume a central role during membrane crossing. These evidences could be helpful in rationalizing results obtained in the present paper. Insertion of glycines spacer between the fluorescent probe and PNT sequence, in fact, does not modify the secondary structure of the peptide in the hydrophobic environment, thus maintaining the penetration efficiency. This efficiency appears unaltered also in the case of PNT-R. This peptide maintains the number of arginine residues of the original PNT sequence but assumes, essentially, an alpha-helical structure in hydrophobic environment, as evidenced by CD spectra (Figure 2). Thus, a correct α-helix/β-sheet ratio, reportedly highly important for CPPs membrane crossing, is not maintained [25] despite probably being compensated for by the increase in hydrophobicity that helps the peptide during membrane core crossing. The distance between the hydrophobic region and the fluorescent cargo, could influence the reported PNT-R behavior. Lastly, any of the synthesized PNT analogues lack, at least, of one arginine residue when compared to the original PNT sequence. The number of arginine residues in PNT and their spatial distribution is probably essential to membrane approaching and insertion. This is the reason why PNT-GG 1, despite sharing similar secondary structure with PNT-GG, shows a reduced membrane penetration ability.

Finally, also the propensity of CPPs to enter corneal epithelium and endothelium cells should be considered. In fact, even if the performance of decapeptides was lower with respect to PNT, they partially maintain the “trans-corneal penetration ability” as testified by their apparent permeability coefficients (except for PNT-R 3), having the same order of magnitude of FAM alone (P*_app_* 0.29 ± 0.08 × 10^−6^ cm/s [6]), a compound characterized by much smaller MW (376.3 g/mol). This suggests that they could have also retained the capability to enter the epithelial cells and thus be useful for an intracorneal targeting, minimizing, with respect to PNT, the delivery to the inner eye structures. However, this should be demonstrated by using different types of approaches, such as imaging technique, since the present ex vivo model does not allow to collect information about peptides distribution within corneal cells (epithelial and endothelial). Despite the relevance of this type of information, required for a deep comprehension of mechanisms characterizing CPPs, the use of the intact isolated tissue, represents a very predictable screening model, useful for selecting peptides able to trans-corneally deliver drugs. In fact, as also described in literature, the results obtained on isolated cells are not always transferrable to membranes and tissues [28]. Due to the complexity of CPPs, it is essential to combine the results deriving from different type of characterization (i.e., circular dichroism) and models (i.e., in vitro, ex vivo, in vivo) to obtain relevant information. Anyway, as always underlined, each combination CPP-cargo represents a unique entity.

Lastly, a very important issue in the use of CPPs is the safety: although safety data for PNT analogues are not available, we can speculate on a significant tolerability, since literature reports PNT lacking in ocular toxicity [14,15].

## 4. Conclusions

In this research work we demonstrated that by reducing the number of amino acids, the resulting PNT analogues only partially retained the parent capability to cross cornea. The high variability of the data, dependent on both tissue and CPPs complexity, did not allow for a clear correlation between the ex vivo behavior and primary and secondary structures.

However, PNT analogues demonstrated a certain potentiality for a corneal targeting and this aspect deserves to be deeply investigated (for instance, using imaging technique), as well as their safety, still an open issue.

## Figures and Tables

**Table 1:** Sequences of PNT and synthesized analogues.

**Peptide**	Sequence ^a^	MW (g/mol) ^b^	Theoretical Charge at pH 7 ^b^
**PNT**	FAM-**R**QI**K**IWFQN**RR**M**K**W**KK**	2604.61	+7
**PNT-GG**	FAM-*GG*-**R**QI**K**IWFQN**RR**M**K**W**KK**	2718.18	+7
**PNT-GG 1**	FAM-*GG*-N**RR**M**K**W**KK**	1617.85	+5
**PNT-GG** **2**	FAM-*GG*-FQN**RR**M**K**W	1636.80	+3
**PNT-GG** **3**	FAM-*GG*-IWFQN**RR**M	1621.79	+2
**PNT-GG** **4**	FAM-*GG*-I**K**IWFQN**R**	1575.74	+2
**PNT-GG** **5**	FAM-*GG*-**R**QI**K**IWFQ	1589.77	+2
**PNT-R**	FAM-*GG*-**KK**W**K**M**RR**NQFWI**K**IQ**R**	2718.18	+7
**PNT-R 1**	FAM-*GG*-QFWI**K**IQ**R**	1589.01	+2
**PNT-R 2**	FAM-*GG*-**R**NQFWI**K**I	1575.68	+2
**PNT-R 3**	FAM-*GG*-M**RR**NQFWI	1622.75	+2
**PNT-R 4**	FAM-*GG*-W**K**M**RR**NQF	1636.81	+3
**PNT-R 5**	FAM-*GG*-**KK**W**K**M**RR**N	1617.85	+5
**PNT-R-FL**	FL-*GG*-**KK**W**K**M**RR**NQFWI**K**IQ**R**	2673.65	+7
**PNT-R-4-FL**	FL-*GG*-W**K**M**RR**NQF	1592.96	+3

^a^ All peptides are amidated at C-terminal. FAM, 5(6) carboxyfluorescein; FL, fluorescein; F, phenylalanine; G, glycine; I, isoleucine; K, lysine; M, methionine; N, asparagine; Q, glutamine; R, arginine; W, tryptophan; ^b^ calculated. Bold letter: positively charged residue. Underlined letter: hydrophobic residue. RRMKWKK: sequence responsible for a productive cell membrane translocation [10].

**Table 2:** Donor concentrations and apparent permeability coefficients calculated using Equation (1).

Peptide	Donor Concentration (µM)	P*_app_* × 10^−6^ (cm/s)	# Replicates
**PNT**	100 ^a^	4.54 ± 2.01	3
**PNT-GG analogues**			
**PNT-GG**	25 ^a^	4.41 ± 2.93	4
**PNT-GG**	250 ^a^	n.d. ^e^	3
**PNT-GG 1**	100 ^a^	1.33 ± 0.11	4
**PNT-GG 2**	100 ^b^	0.55 ± 0.57	3
**PNT-GG 3**	100 ^b^	0.44 ± 0.50	3
**PNT-GG 4**	100 ^b^	0.34 ± 0.14	3
**PNT-GG 5**	100 ^b^	0.24 ± 0.40 ^d^	3
	100 ^c^	0.50 ± 0.70 ^d^	3
**PNT-R analogues**			
**PNT-R**	25 ^a^	7.08 ± 6.66	4
**PNT-R**	250 ^a^	n.d. ^e^	3
**PNT-R 1**	250 ^a^	0.93 ± 0.53	4
**PNT-R 2**	250 ^a^	0.23 ± 0.16	3
**PNT-R 3**	250 ^a^	0.002 ± 0.004	3
**PNT-R 4**	250 ^a^	0.13 ± 0.07	4
**PNT-R 5**	250 ^a^	0.05 ± 0.01	3

^a^ donor and receiving solutions: PBS pH 7.4; ^b^ donor and receiving solutions: PBS+BSA 0.1% pH 7.4; ^c^ donor solution: PBS pH 7.4; receiving solution: PBS+BSA 0.1% pH 7.4; ^d^ *p* = 0.6; ^e^ n.d., not determined.

## Supplementary Materials

The following are available online at https://www.mdpi.com/1999-4923/12/8/728/s1, Table S1: Analytical data of synthesized peptides; Table S2: Range of calibration curves, RSD% (relative standard deviation %) and ER% (relative error %); Figures S1–S15: Mass spectrometry and HPLC of peptides used in the study.
