# Supplementary material for: Synthesis and Ex Vivo Trans-Corneal Permeation of Penetratin Analogues as Ophthalmic Carriers: Preliminary Results
Source: Pharmaceutics. 2020 Aug 3;12(8):728. doi: 10.3390/pharmaceutics12080728 (PMC7466059; doi:10.3390/pharmaceutics12080728)
Supplement: Supplementary file 1 [file pharmaceutics-12-00728-s001.pdf]

pharmaceutics

Supplementary Materials: Synthesis and Ex Vivo Trans-Corneal Permeation of Penetratin Analogues as Ophthalmic Carriers: Preliminary Results

Silvia Pescina, Marina Sala, Maria Carmina Scala, Patrizia Santi, Cristina Padula, Pietro Campiglia, Carmine Ostacolo and Sara Nicoli

Table S1. Analytical data of synthesized peptides.

Peptide	Sequence	HPLC	ESI-MS		
		k′[a]	Precursor ion (m/z)[b]	Product ion (m/z)	z
**PNT**	FAM-RQIKIWFQNRRMKWKK	12.89	2604.61	868.83<br>651.85<br>521.69	3<br>4<br>5
**PNT-GG**	FAM-GG-RQIKIWFQNRRMKWKK	7.33	2718.18	906.82<br>680.36<br>544.48	3<br>4<br>5
**PNT-GG 1**	FAM-GG-NRRMKWKK	9.02	1617.85	809.35<br>539.89<br>405.17	2<br>3<br>4
**PNT-GG 2**	FAM-GG-FQNRRMKW	8.05	1636.80	818.84<br>546.22	2<br>3
**PNT-GG 3**	FAM-GG-IWFQNRRM	7.87	1621.79	811.31<br>541.21	2<br>3
**PNT-GG 4**	FAM-GG-IKIWFQNR	11.51	1575.74	788.26<br>525.85	2<br>3
**PNT-GG 5**	FAM-GG-RQIKIWFQ	9.06	1589.77	1590.20	1
**PNT-R**	FAM-GG-KKWKMRRNQFWIKIQR	7.31	2718.18	906.70<br>680.70<br>544.40	3<br>4<br>5
**PNT-R 1**	FAM-GG-QFWIKIQR	9.10	1589.01	1590.22<br>796.13	1<br>2
**PNT-R 2**	FAM-GG-RNQFWIKI	11.45	1575.68	1575.62<br>789.08	1<br>2
**PNT-R 3**	FAM-GG-MRRNQFWI	7.84	1622.75	1622.72<br>812.21	1<br>2
**PNT-R 4**	FAM-GG-WKMRRNQF	8.01	1636.81	819.20	2
**PNT-R 5**	FAM-GG-KKWKMRRN	8.99	1617.85	809.23<br>540.01<br>405.22	2<br>3<br>4
**PNT-R-FL**	FL-GG-KKWKMRRNQFWIKIQR	12.48	2673.65	1337.60<br>892.10	2<br>3
**PNT-R 4-FL**<br>**PNT-R-4-FL 4**	FL-GG-WKMRRNQF	6.77	1592.96	1593.23	1

[a] k′=[(peptide retention time-solvent retention time)/solvent retention time]. [b] calculated.

Table S2. Range of calibration curves, RSD% (relative standard deviation %) and ER% (relative error %).

Peptide	Linearity range (nM)	RSD%	ER%
**PNT**	10-100	5	10
**PNT-GG**	10-100	10	15
**PNT-GG 1**	100-500	10	15
**PNT-GG 2**	5-100	10	15
**PNT-GG 3**	1-100	5	5
**PNT-GG 4**	10-100	10	15
**PNT-GG 5**	5-100	10	15
**PNT-R**	25-100	10	15
**PNT-R 1**	10-100	10	15
**PNT-R 2**	10-50	10	15
**PNT-R 3**	0.5-5	10	15
**PNT-R 4**	5-100	10	10
**PNT-R 5**	0.5-25	10	15

Supplementary figures of Mass spectrometry and HPLC of peptides used in the study

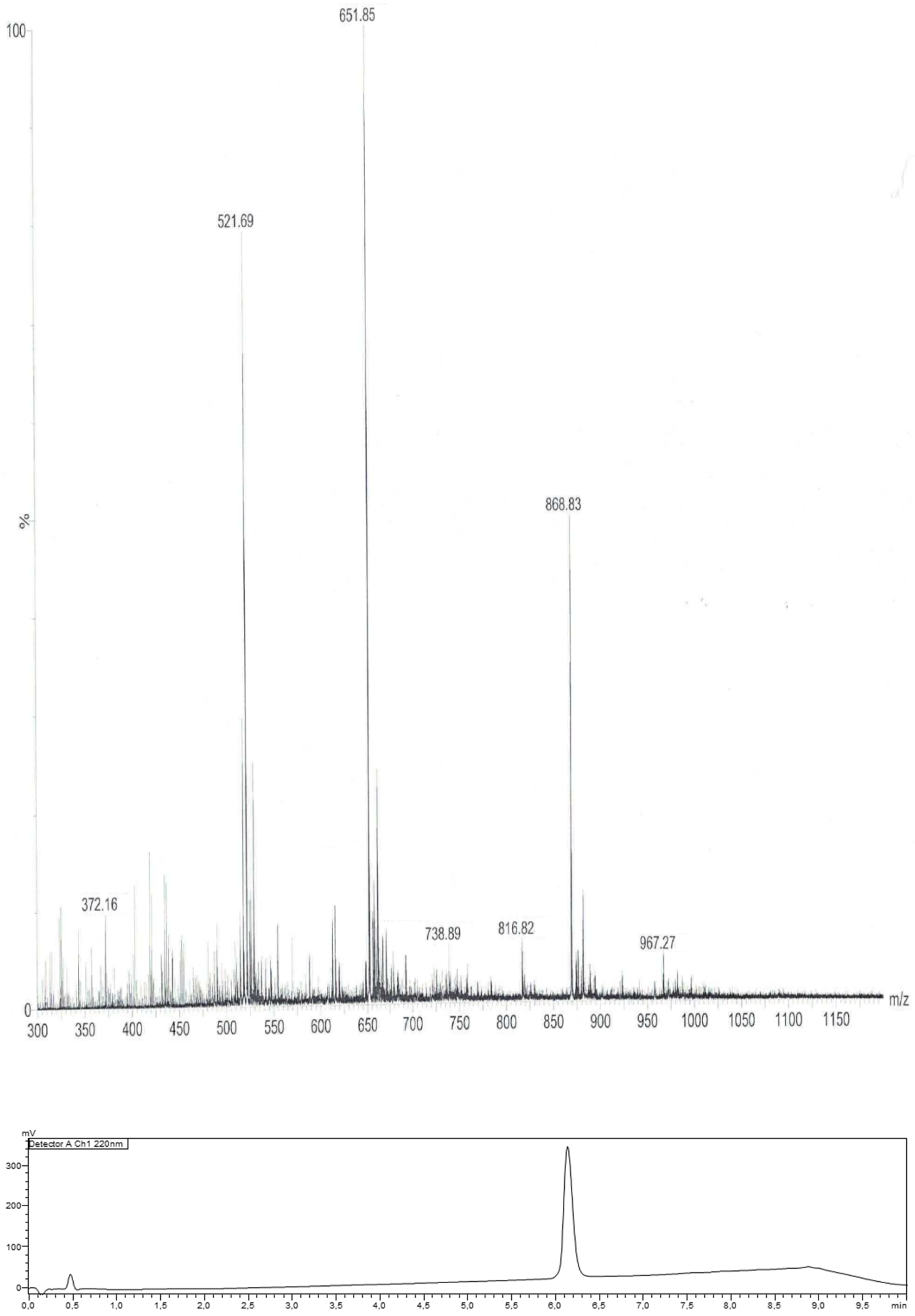

Figure S1. ESI-MS of PNT and analytical HPLC trace at 220 nm.

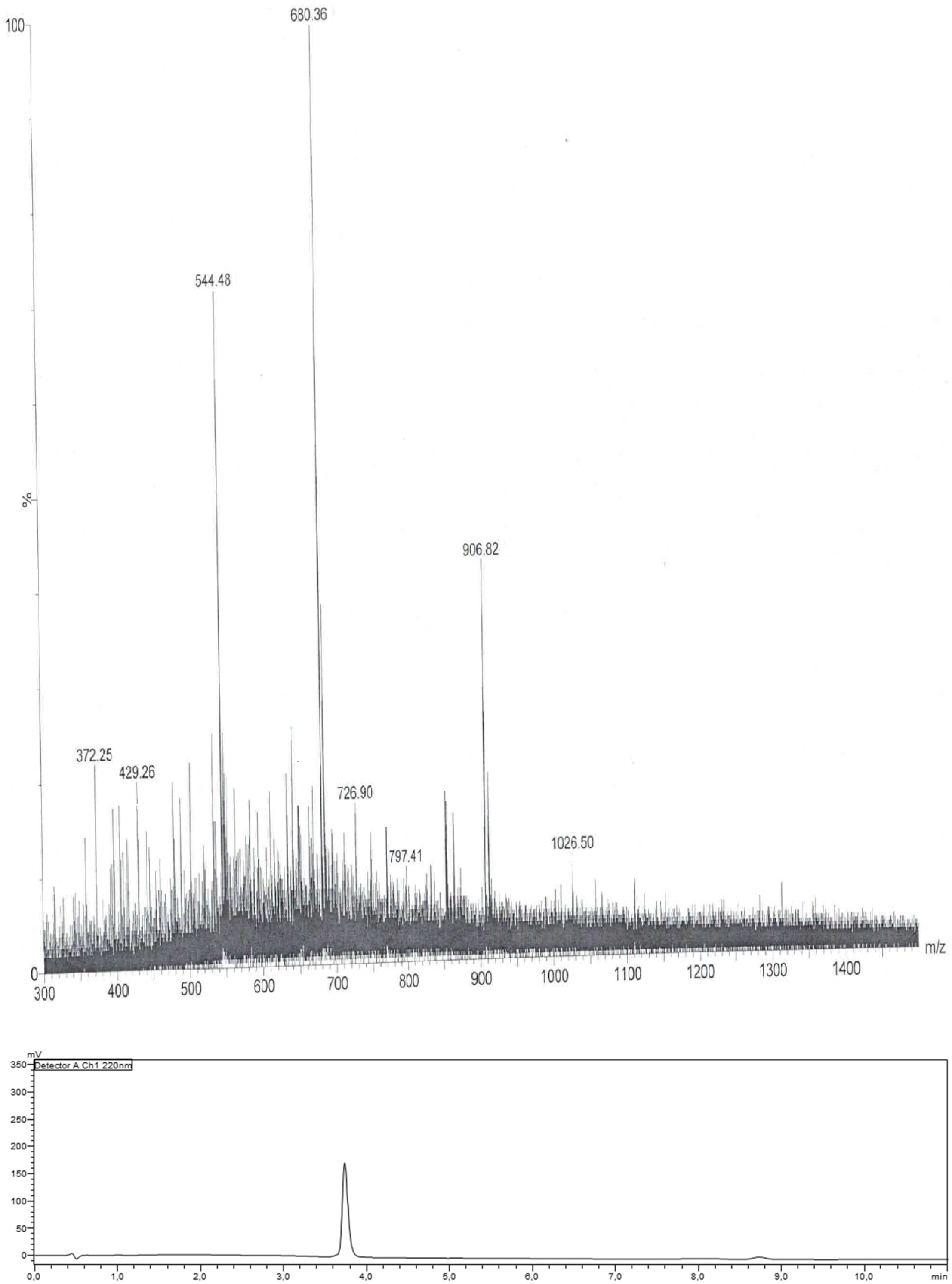

Figure S2. ESI-MS of PNT-GG and analytical HPLC trace at 220 nm.

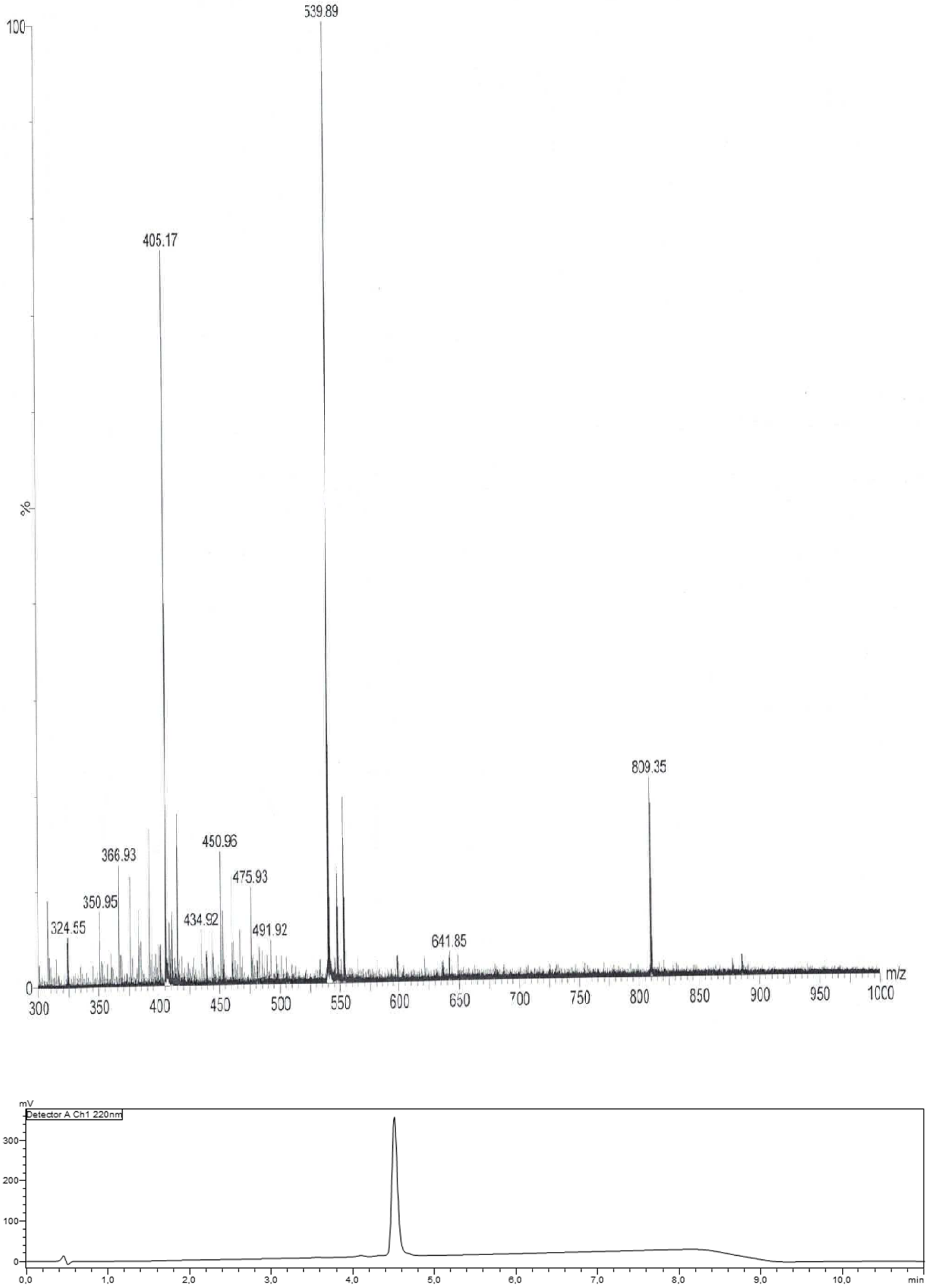

Figure S3. ESI-MS of PNT-GG 1 and analytical HPLC trace at 220 nm.

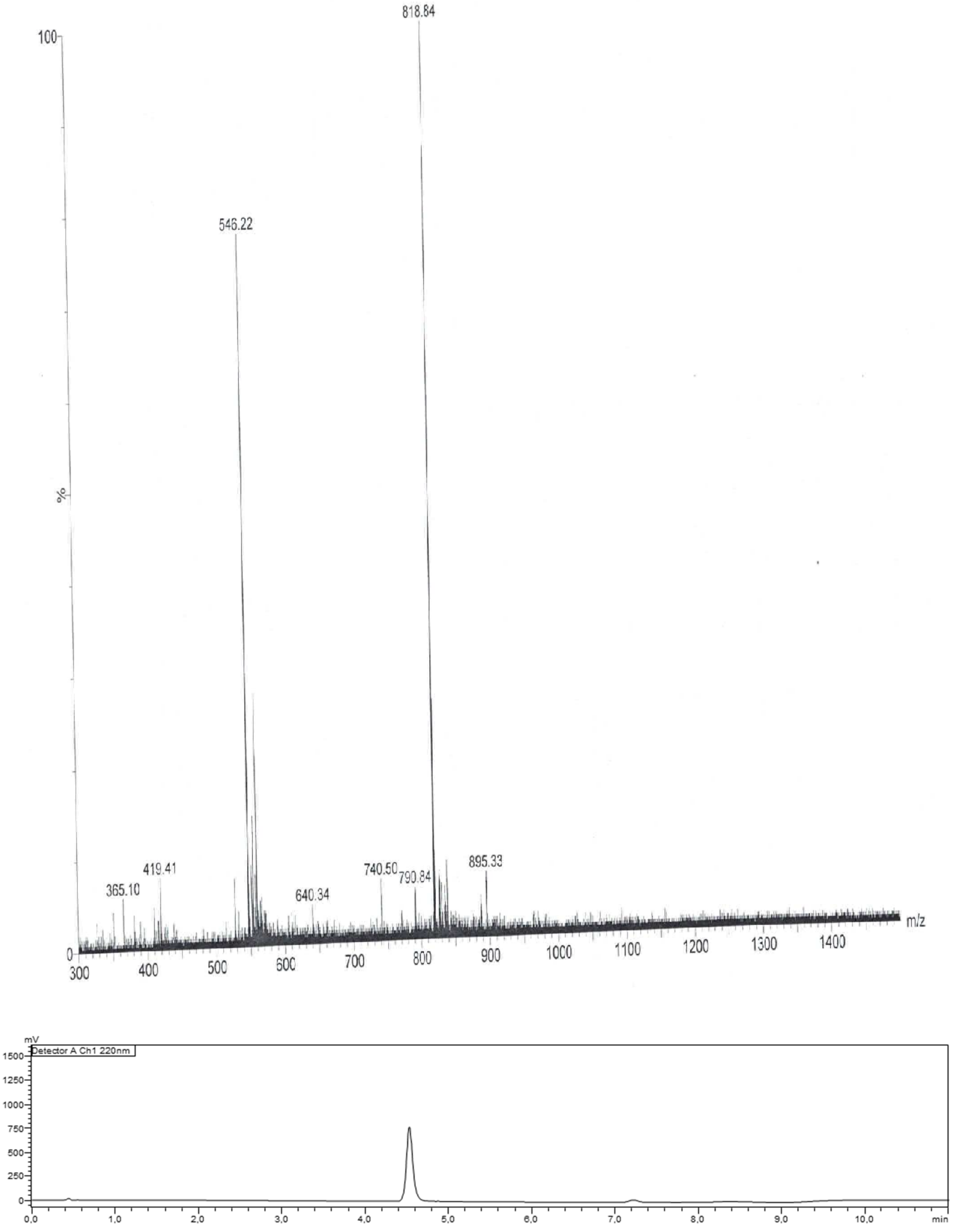

Figure S4. ESI-MS of PNT-GG 2 and analytical HPLC trace at 220 nm.

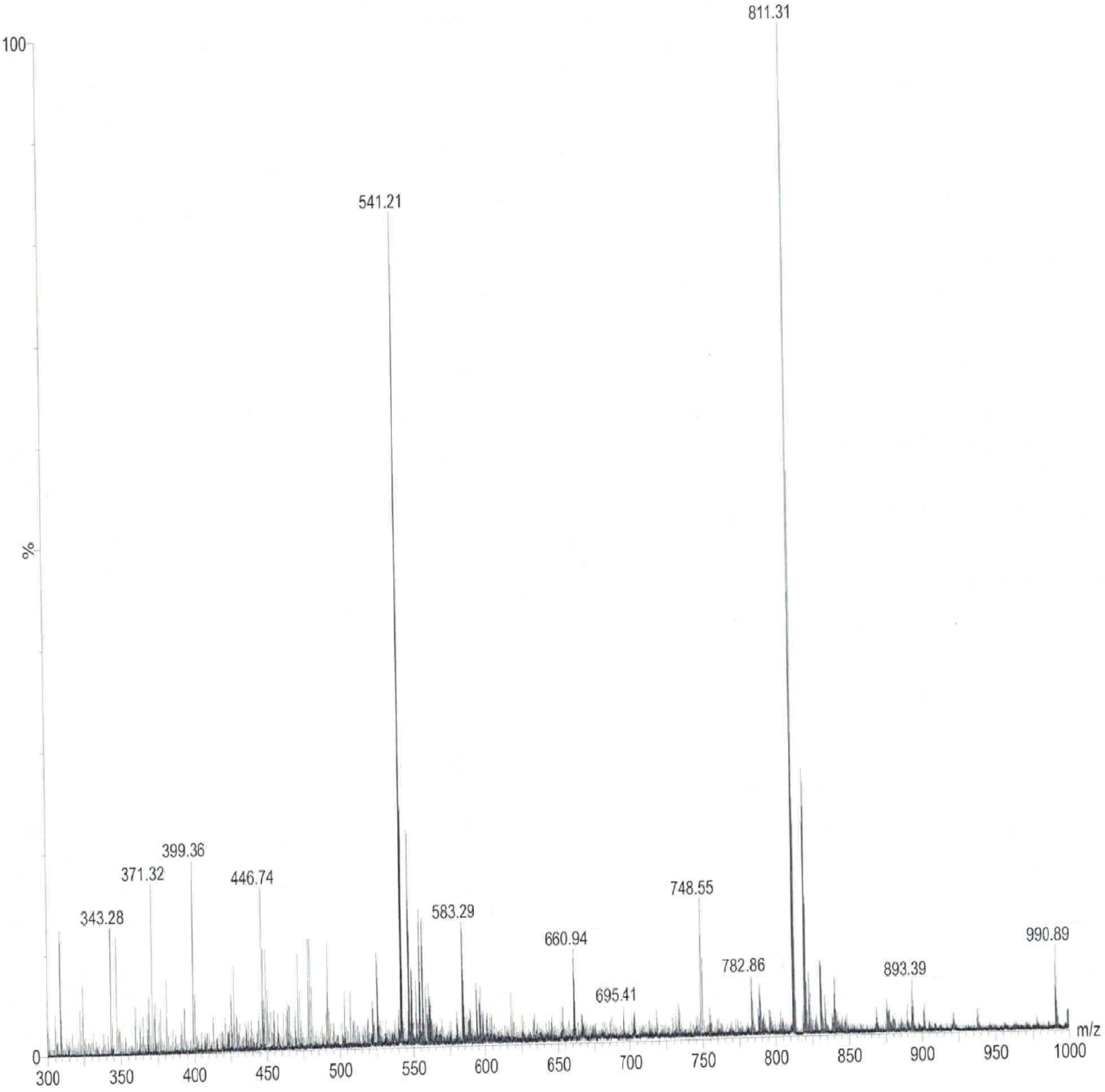

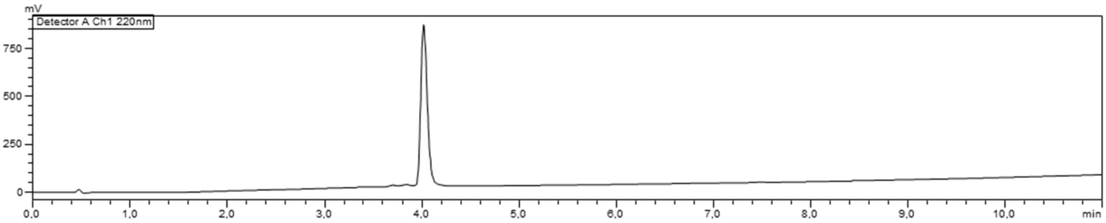

Figure S5. ESI-MS of PNT-GG 3 and analytical HPLC trace at 220 nm.

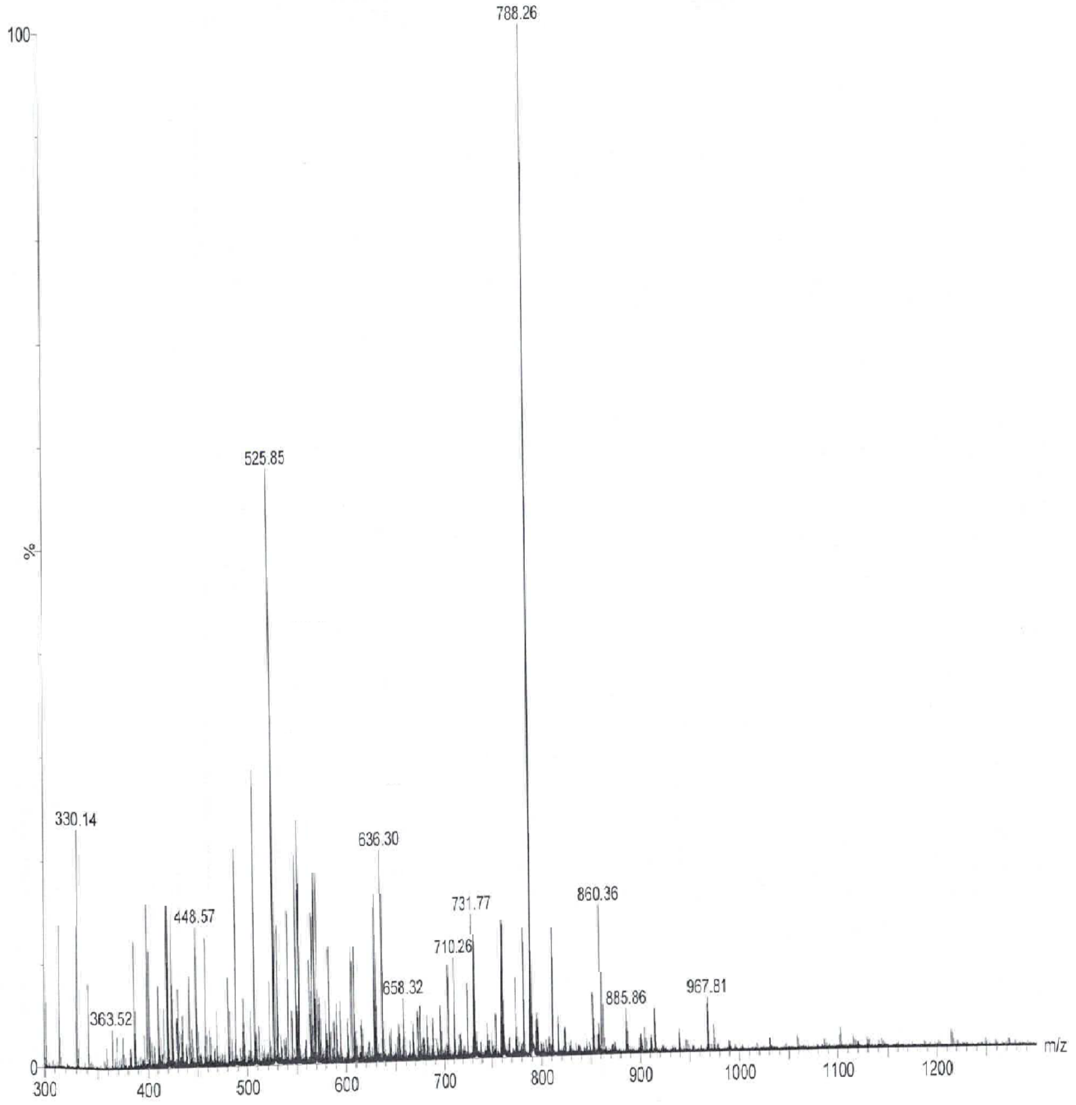

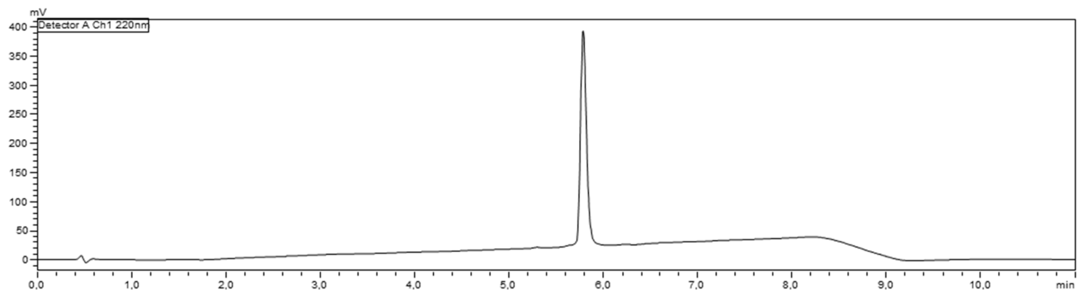

Figure S6. ESI-MS of PNT-GG 4 and analytical HPLC trace at 220 nm.

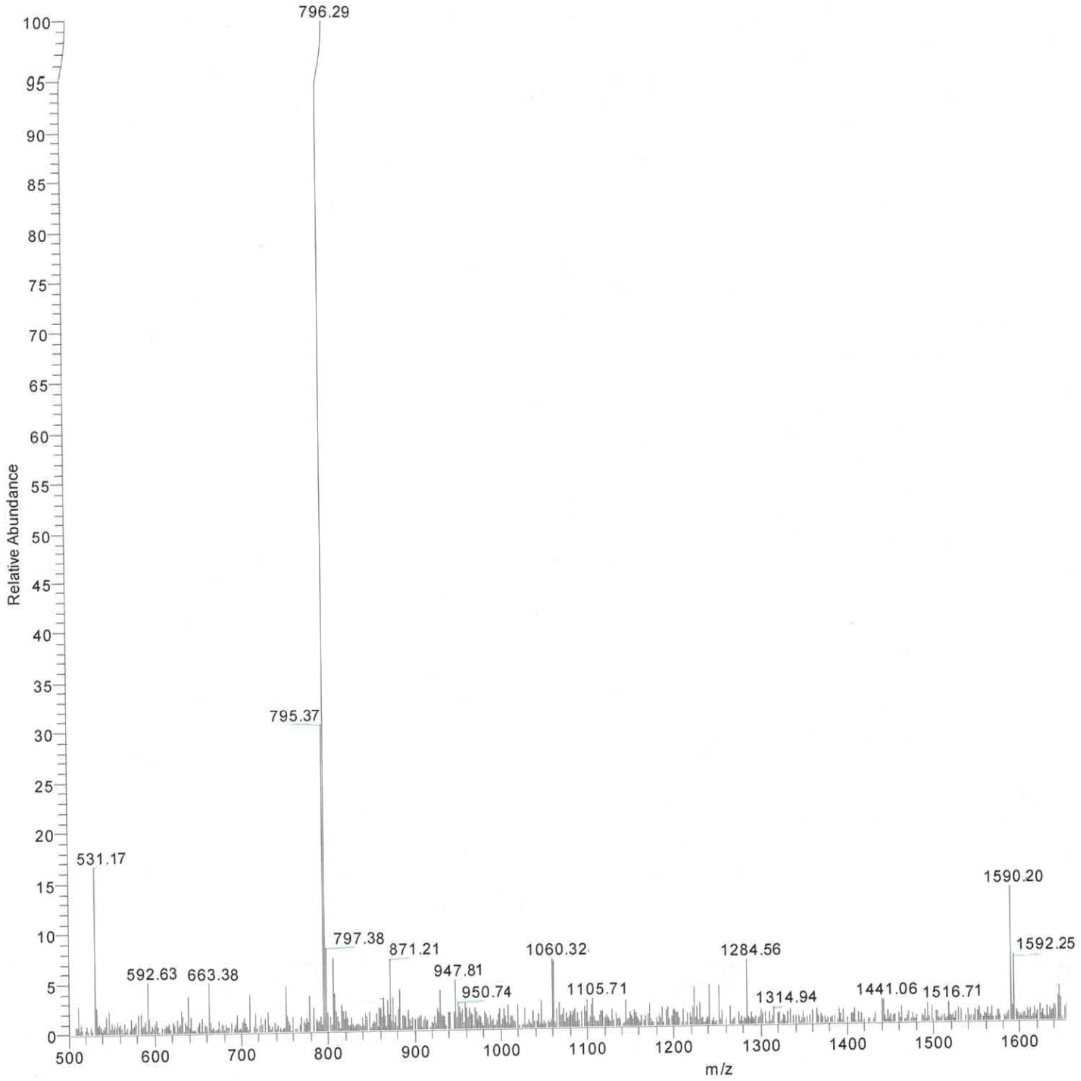

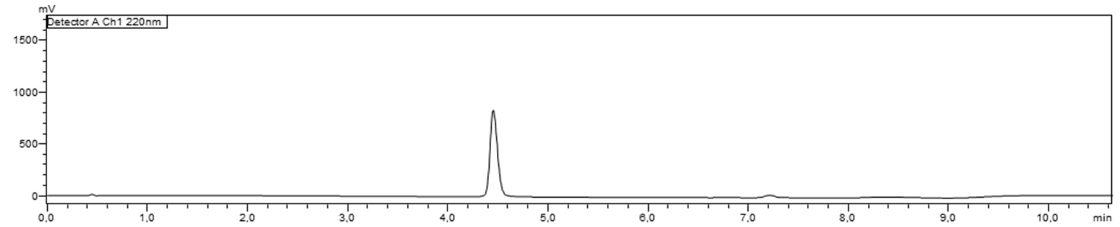

Figure S7. ESI-MS of PNT-GG 5 and analytical HPLC trace at 220 nm.

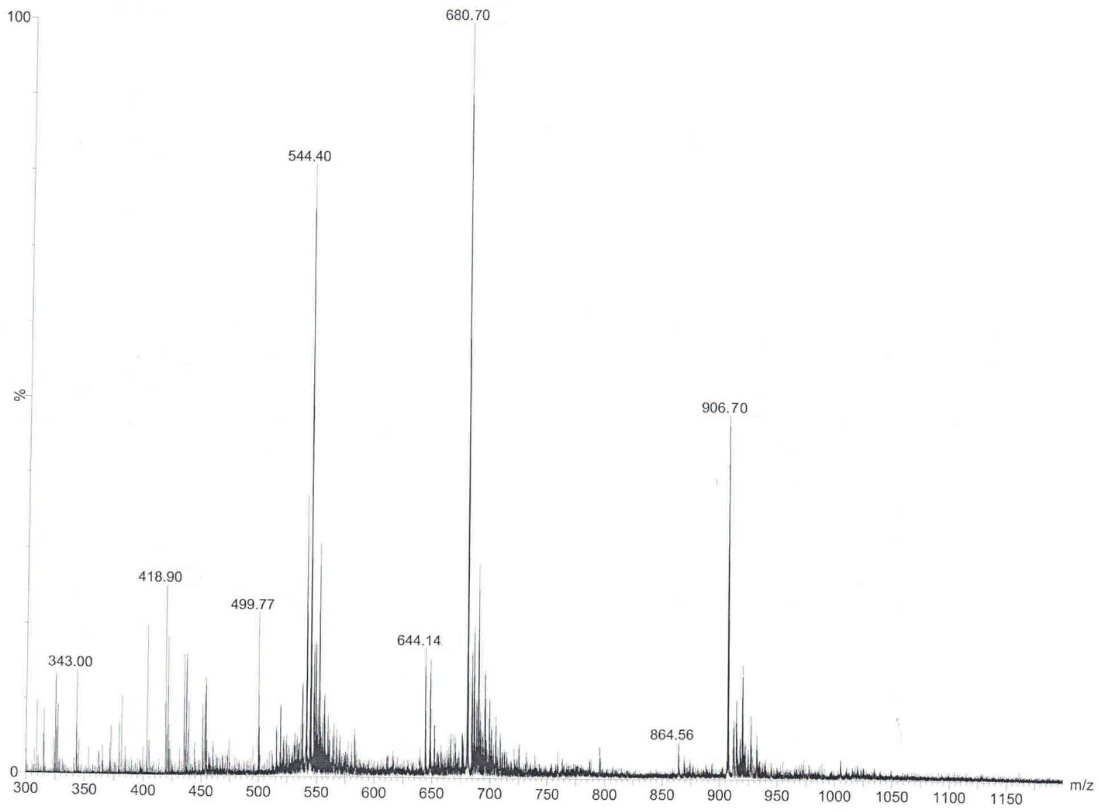

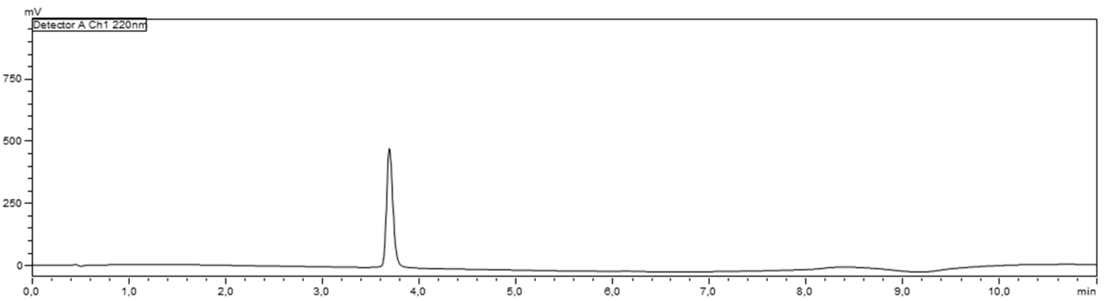

Figure S8. ESI-MS of PNT-R and analytical HPLC trace at 220 nm.

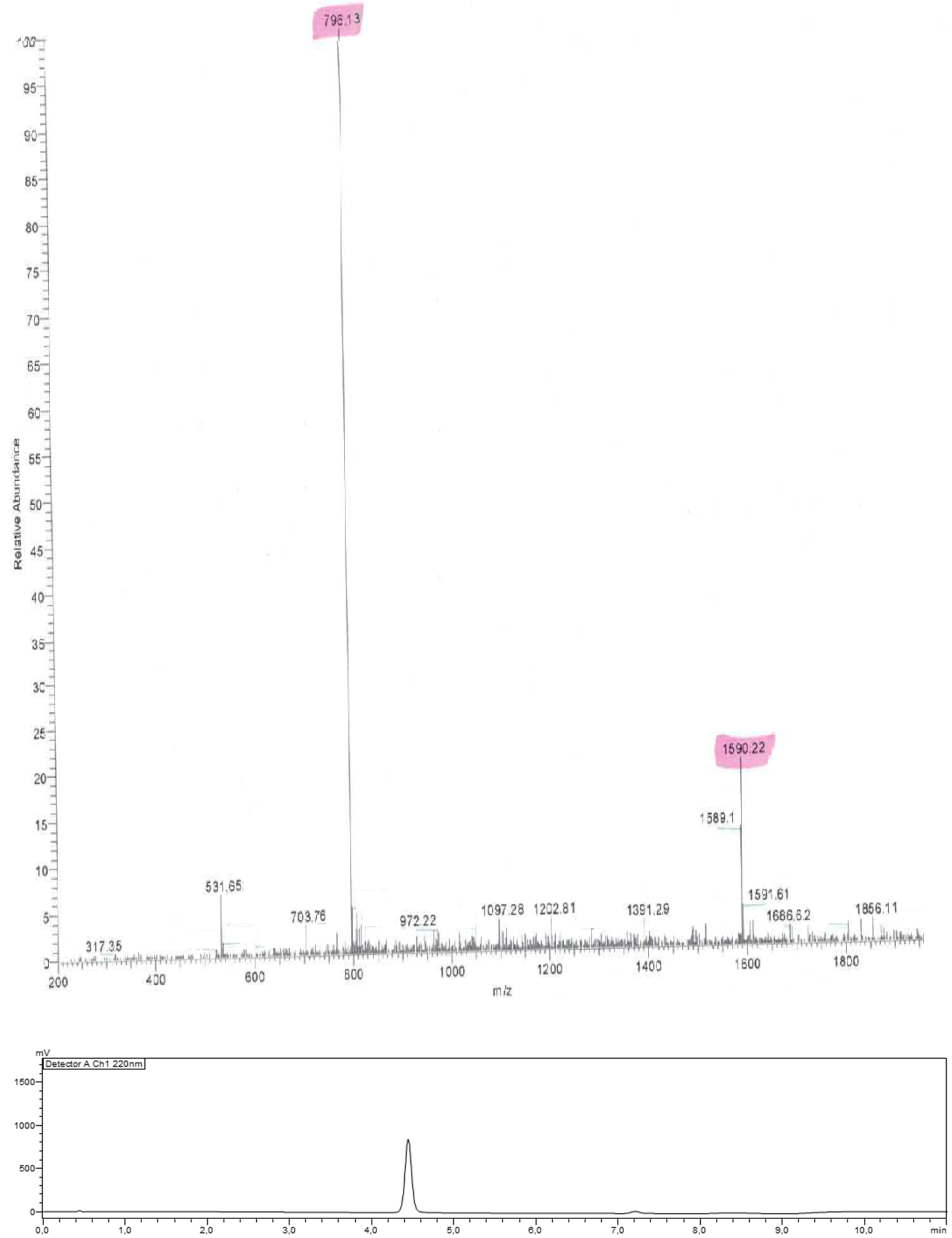

Figure S9. ESI-MS of PNT-R 1 and analytical HPLC trace at 220 nm.

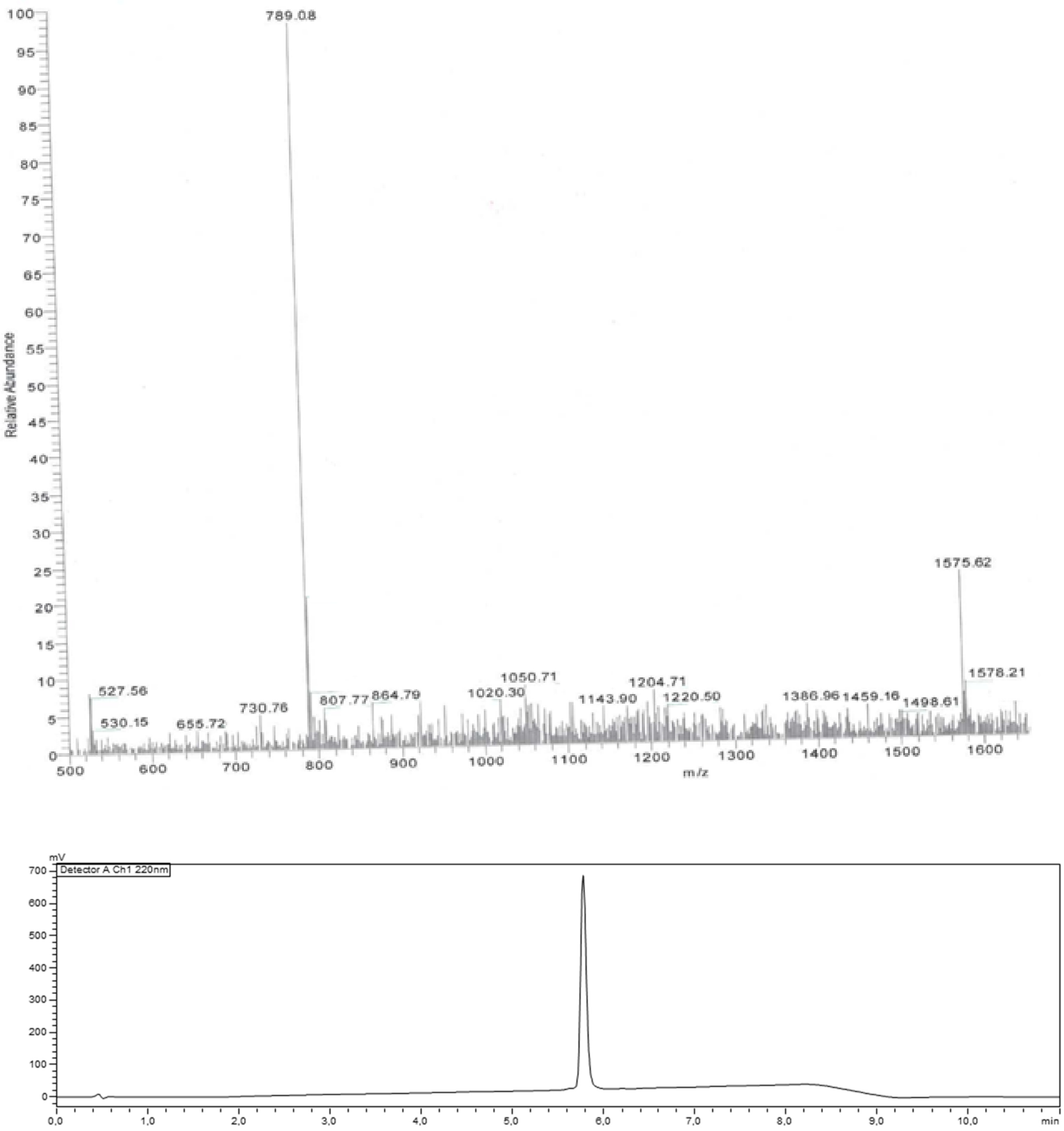

Figure S10. ESI-MS of PNT-R 2 and analytical HPLC trace at 220 nm.

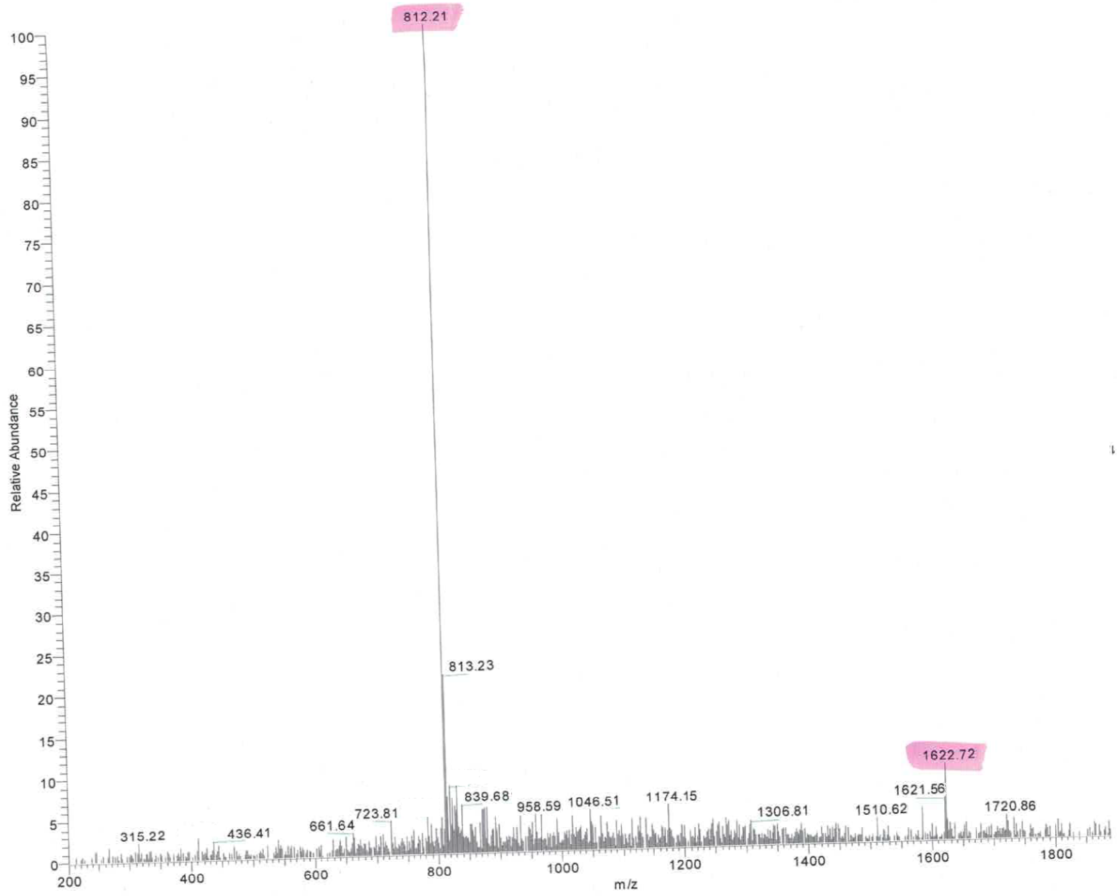

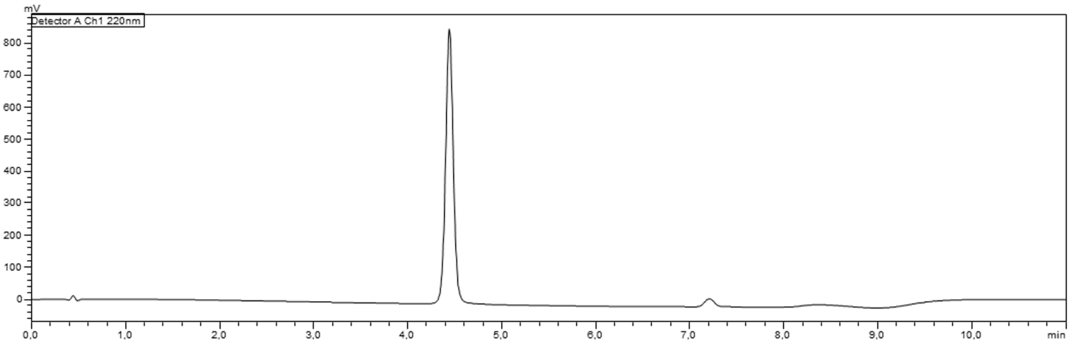

Figure S11. ESI-MS of PNT-R 3 and analytical HPLC trace at 220 nm.

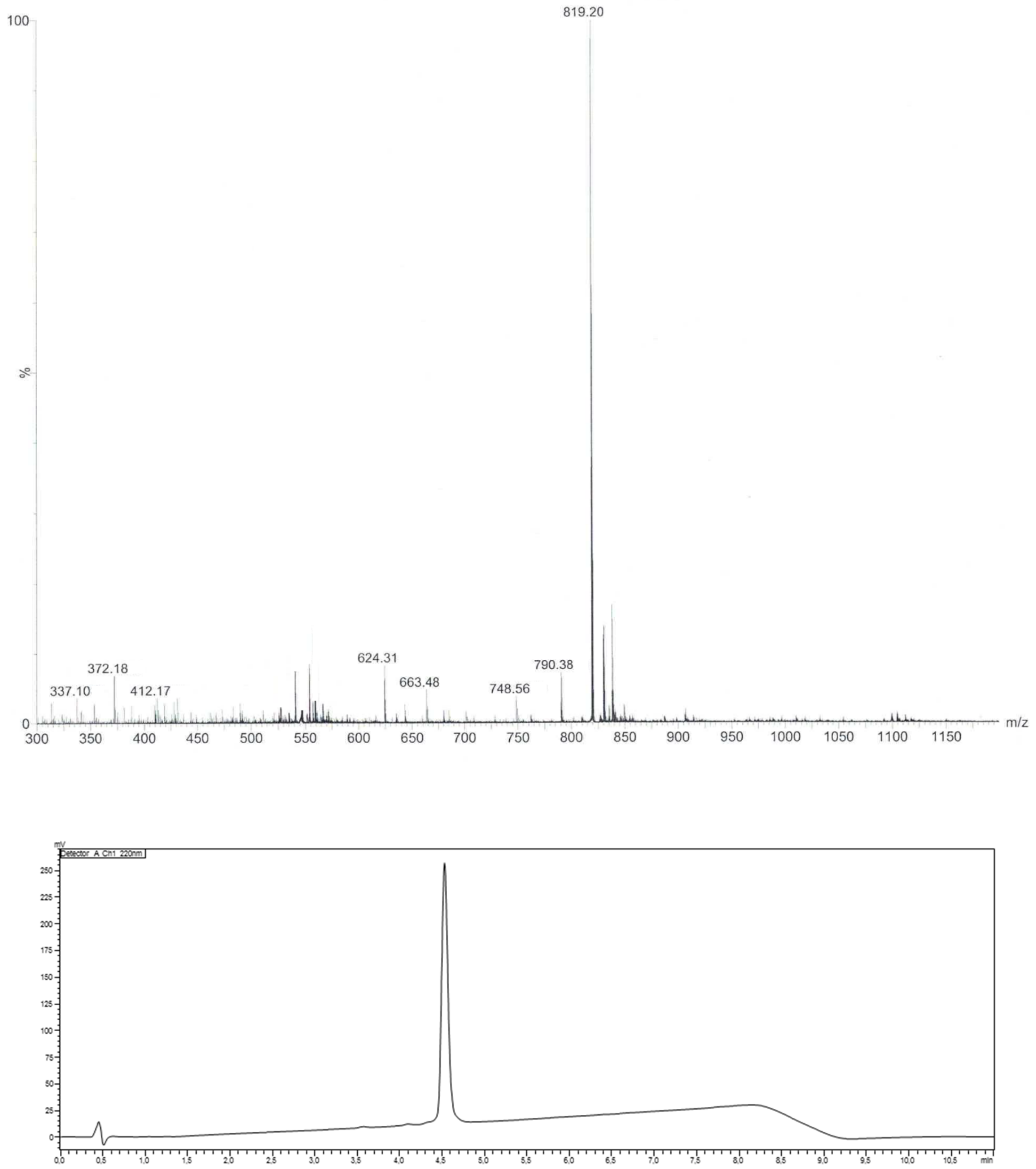

Figure S12. ESI-MS of PNT-R 4 and analytical HPLC trace at 220 nm.

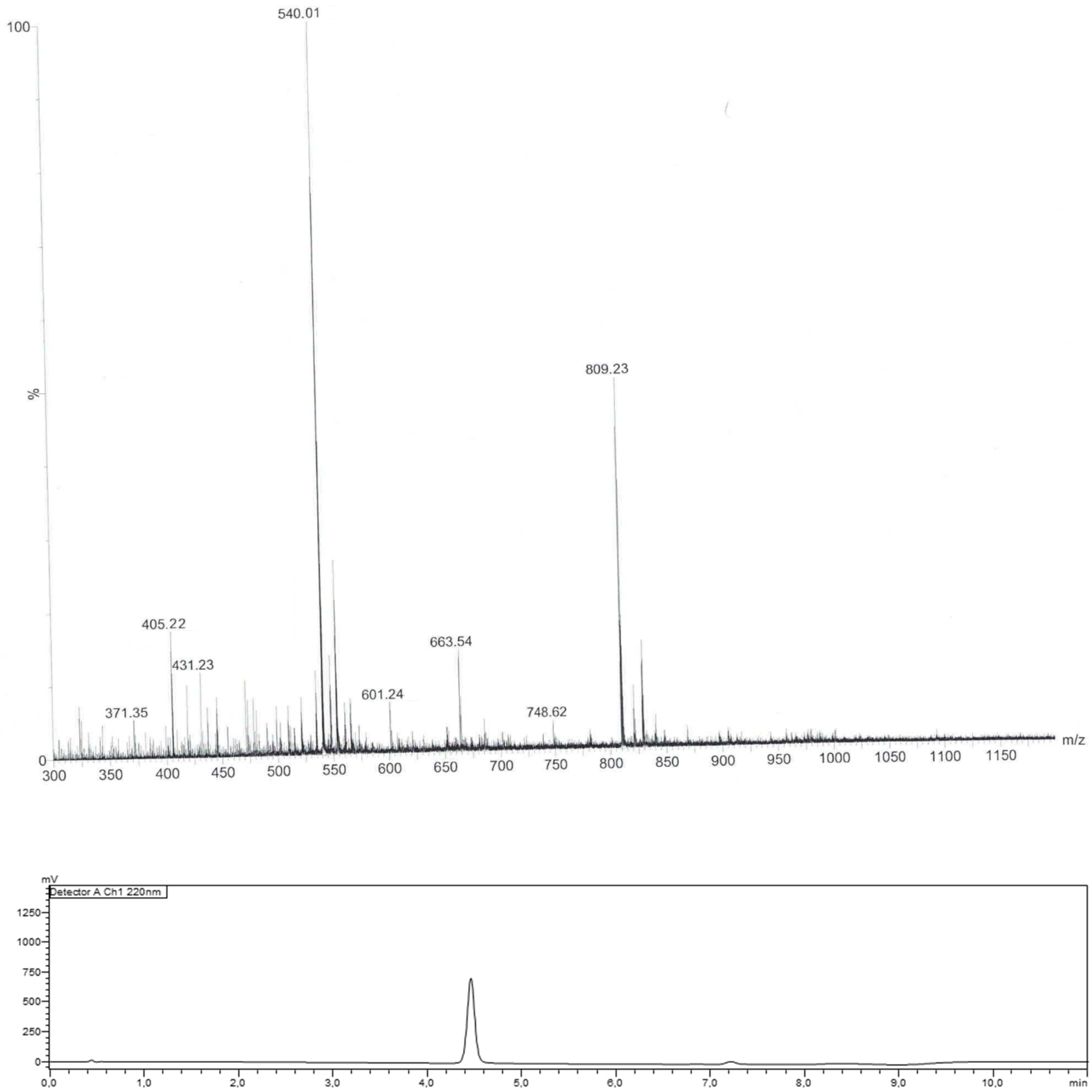

Figure S13. ESI-MS of PNT-R 5 and analytical HPLC trace at 220 nm.

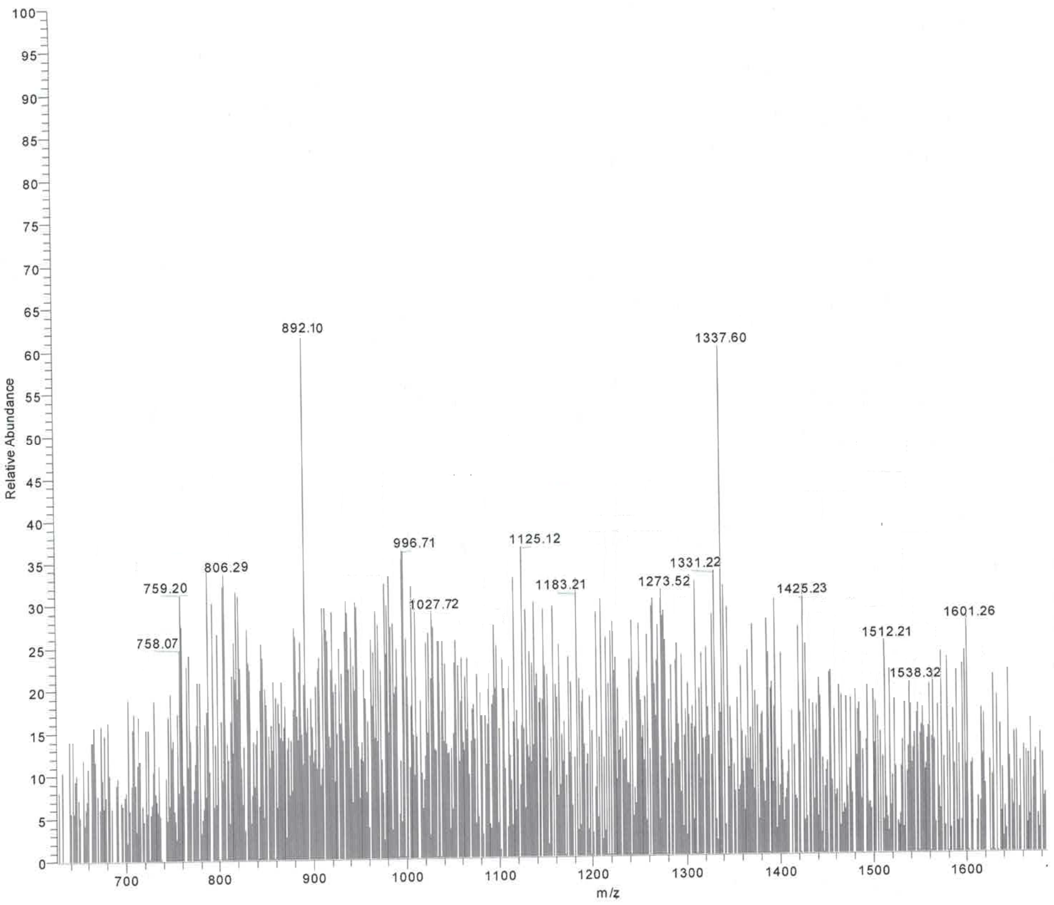

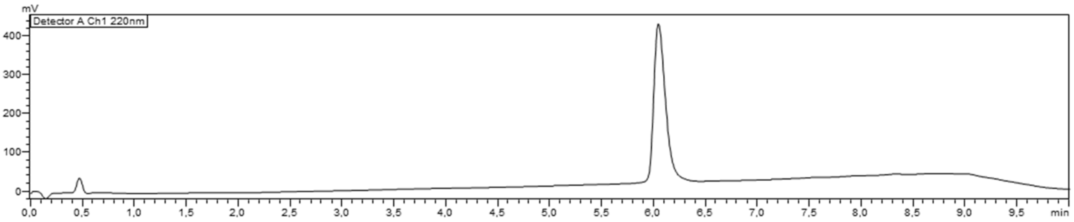

Figure S14. ESI-MS of PNT-R-FL and analytical HPLC trace at 220 nm.

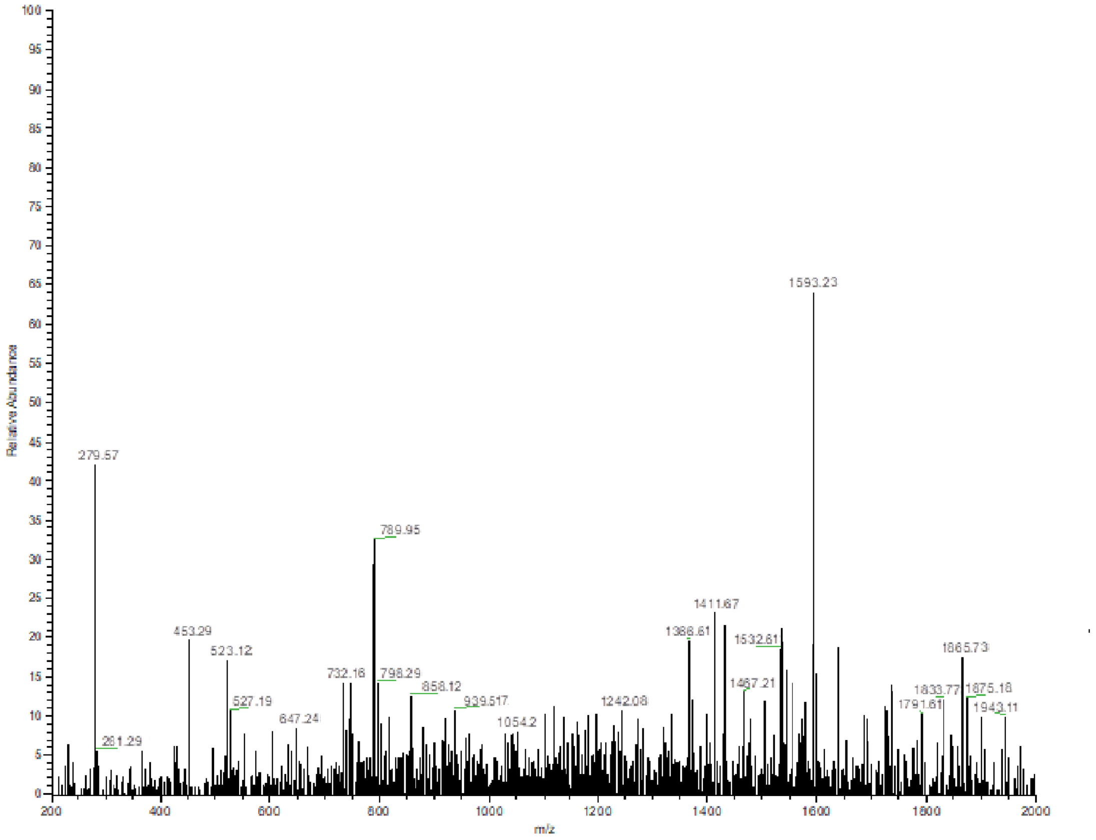

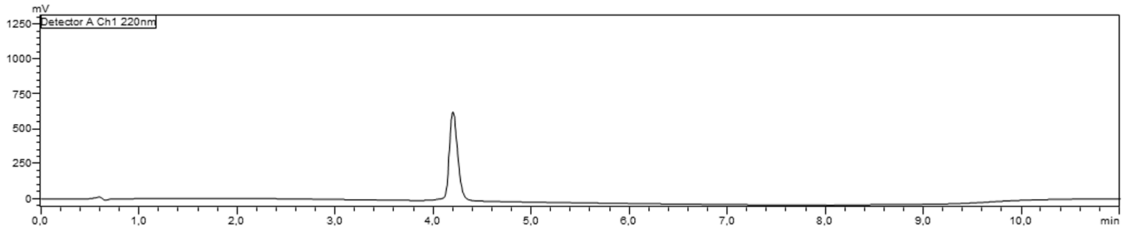

Figure S15. ESI-MS of PNT-R 4-FL and analytical HPLC trace at 220 nm.
